# Is the incidence of hip fracture increasing among older men in England?

**DOI:** 10.1136/jech-2015-207114

**Published:** 2016-01-21

**Authors:** Jenny Neuburger, Robert Wakeman

**Affiliations:** 1 Department of Health Services Research & Policy, London School of Hygiene & Tropical Medicine, London, UK; 2 Basildon & Thurrock University Hospitals NHS Foundation Trust, Basildon, Essex, UK

**Keywords:** FRACTURES, Epidemiology of ageing, GENDER

The prevention of hip fracture has been a long-term goal for healthcare in England.^1^ Over the past decade, there have been numerous initiatives aimed at reducing the risk of fracture among frail older people.^2^ We examined trends in the incidence of hip fracture over the last decade and noticed a difference between women and men. While rates have decreased among women, they have increased among men.

We calculated age-specific incidence separately for women and men for each calendar year from 2003 to 2013. We combined two datasets: (1) Hospital Episode Statistics were used to identify the number of people admitted to hospital with hip fracture;^3^ and (2) Office for National Statistics’ mid-year population estimates were used as denominators.^4^ We calculated population incidence rates for three age groups: 60–74; 75–84; and 85 years and older.

**Figure 1:**
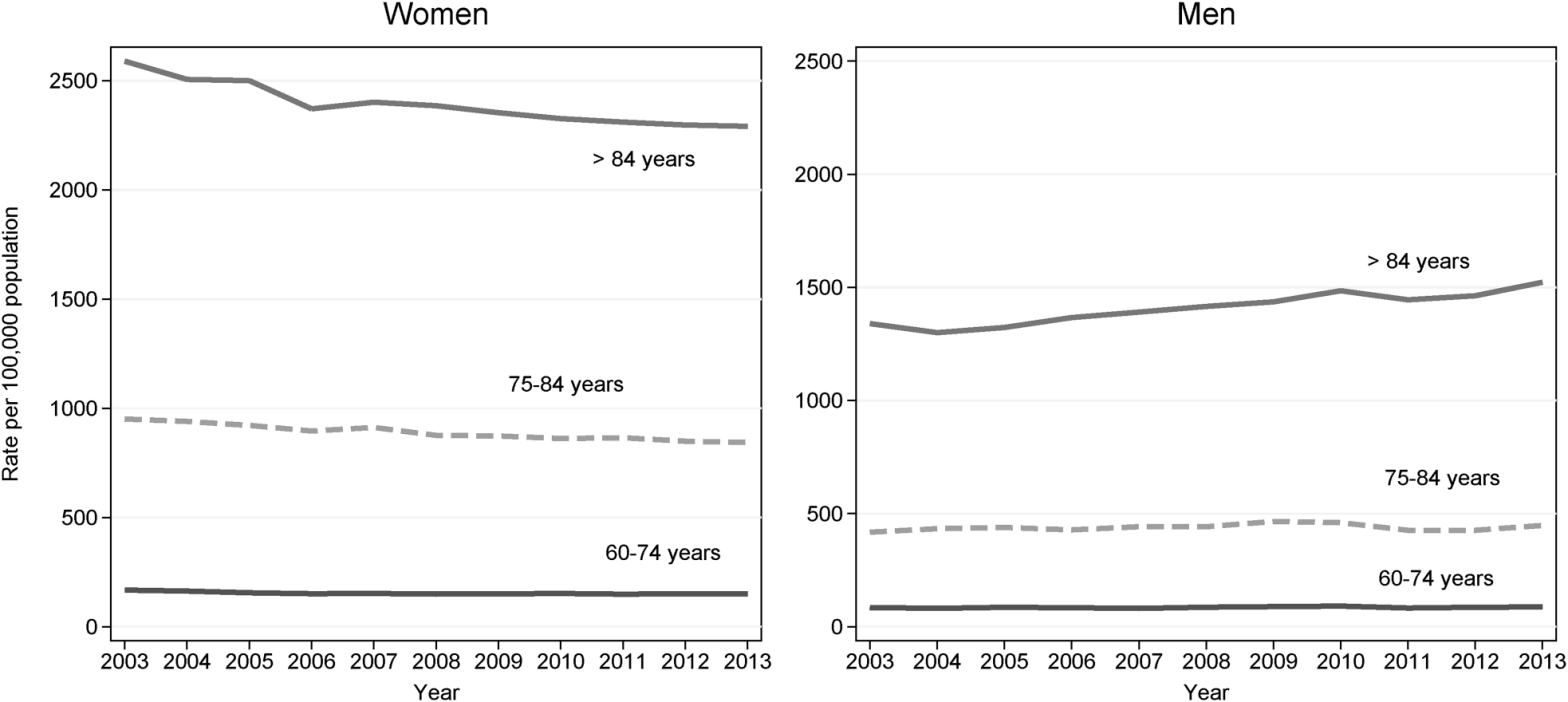
Incidence of hip fracture (rates per 100 000) in women and men in England, 2003–2013.

Since 2003, the number of people admitted with a first hip fracture in England has risen from 50 495 to 55 353, and the proportion of men in this group increased from 21.9% to 28.6%. The population incidence of hip fracture in women and men up to the age of 75 years has remained stable, while the rate in women aged 75 and over has decreased. In contrast, incidence among older men (85 years and older) has increased (figure 1).

Other investigators have shown a reduction in the incidence of hip fracture in the USA for women and men.^5^ In England, the gender difference in trends may be partly due to the perception that osteoporosis is a woman's disease. As a result, even if men have a signal fracture prior to their first hip fracture, referral for bone health assessment might be less common. There may be gender differences in use and effectiveness of bone health medication.

The increase in incidence among older men could also be due to improved survival, leading to a higher proportion at risk of falls and fractures. Yet another factor could be the impact of androgen deprivation therapy for prostate cancer on bone quality.^6^

Commissioners in England should be aware of this increasing incidence of hip fracture among men and should ensure that men have parity of access to fracture liaison services.
